# Organizational monitoring of patient blood management implementation: Results of an Italian national survey

**DOI:** 10.1111/vox.70267

**Published:** 2026-05-15

**Authors:** Vanessa Agostini, Francesca Masiello, Stefania Vaglio, Eva Veropalumbo, Ursula La Rocca, Simonetta Pupella, Vincenzo De Angelis, Luciana Teofili

**Affiliations:** ^1^ Transfusion Medicine Department IRCCS‐Ospedale Policlinico San Martino Genoa Italy; ^2^ National Blood Centre Italian National Institute of Health Rome Italy; ^3^ Department of Molecular Medicine Sapienza University Rome Italy; ^4^ Department of Laboratory and Transfusion Medicine Misericordia Hospital, Azienda Usl Toscana Sudest Grosseto Italy

**Keywords:** anaemia, blood transfusion, operational procedures, patient blood management, transfusion medicine

## Abstract

**Background and Objectives:**

Patient blood management (PBM) as a standard‐of‐care approach, sustained by substantial scientific literature, is now widely supported by national and international recommendations and guidelines. Scientific evidence shows that PBM is a strategic tool for improving clinical outcomes, quality of life and patient safety, as well as reducing inappropriate blood component consumption and lowering healthcare costs.

**Materials and Methods:**

Following up the previous monitoring results (a report on 2018 data) and considering new international policy documents such as World Health Organization (WHO) policy brief, the National Blood Centre (Centro Nazionale Sangue [CNS]) has developed a new survey in 2021 aimed to investigate mainly the organizational implementation of preoperative anaemia management of PBM.

**Results:**

Project adherence was 70%, with 14 responding transfusion services (TSs) out of 20 involved, distributed throughout the country. TSs reporting the presence of a corporate procedure on PBM were 35.7%. Only 50% of TSs reported preparing an annual report on PBM, and of these, 90% analysed the number of anaemic patients undergoing surgery who received iron therapy in relation to the number of anaemic patients identified. All TSs conduct an analysis of the reduction in blood component consumption, but only 2 out of 14 TSs, from two different regions, conduct annual audits on PBM.

**Conclusion:**

This second survey made it possible to compare the main critical issues identified in the various local areas and to develop a set of recommendations for the management of an advanced PBM system to be applied throughout the country.

**Highlights**

- Although transfusion services (TSs) attempt to implement patient blood management (PBM), implementation remains inconsistent and not yet widespread.
- The primary indicator in the annual PBM report is the number of anaemic patients who are candidates for surgery and who receive iron therapy compared to the number of anaemic patients identified.
- About the PBM‐related costs, the TSs only considered iron therapy, but they should include a comparison with the period prior to implementation—in particular, costs related to the anaesthesiology assessment clinic, costs related to preoperative assessment investigations and costs related to drug administration.

## INTRODUCTION

‘Patient blood management (PBM) is a patient‐centred, systematic, evidence‐based approach to improve patient outcomes by managing and preserving a patient's own blood, while promoting patient safety and empowerment’ [1]. This global definition of PBM highlights a multidisciplinary application of evidence‐based medical and surgical concepts to appropriately diagnose and treat preoperative anaemia, to minimize blood loss during surgeries, procedures and laboratory draws and to guide patients towards appropriate treatment. In the last decade, many countries have introduced PBM programmes to reduce the use of blood products and improve tolerance to anaemia; in addition, various PBM initiatives are being developed and implemented around the world [1].

The World Health Organization (WHO) states that delaying PBM implementation increases morbidity and mortality. Therefore, WHO urges its 194 Member States to act quickly with their ministries and departments of health to create a national PBM policy, establish the necessary governance to improve population health and reduce healthcare expenditure [2].

The National Blood Centre (Centro Nazionale Sangue [CNS]) is the Italian government authority responsible for coordinating the transfusion system and promoting PBM in line with WHO indications. CNS has set up various projects to promote PBM, starting with a national project aimed at promoting the first pilot applications of PBM in elective orthopaedic surgery in adults [3].

The Decree of the Minister of Health of 2 November 2015 updated the quality and safety requirements for blood and blood components across all stages of the process, incorporating recent scientific developments and aligning with European regulations [4]. This Decree provides to define detailed PBM programmes throughout the country to prepare patients for scheduled surgical treatments, based on specific guidelines issued by the CNS [5].

In 2018, CNS launched a project aimed at assessing the level of PBM implementation across Italy through a dedicated survey [6, 7], in line with proposals elaborated in the recent international scientific literature [8]. This first national survey highlighted the widespread adoption of pathways for the diagnosis and treatment of anaemia in the perioperative setting, the effective use of anaemia screening programmes, the use of single unit policy in line with national guideline recommendations and poor reporting of clinical outcomes and indicators.

For this reason, this paper is based on an in‐depth analysis of the organizational elements related to PBM focused on the first pillar, which aims to reduce the need for transfusions through early detection and targeted treatment of anaemia. The analysis of data obtained from a second dedicated survey aims to define implementation models for PBM, supported by key performance indicators (KPIs) for systematic monitoring.

## MATERIALS AND METHODS

A survey on the organizational aspects of PBM involved 20 transfusion services (TSs) across the country; it was conducted in September 2021 and is presented as Supporting Information S1.

The survey was designed by the working group for the development of transfusion activities and the improvement of the transfusion network established within the CNS; the possible responses were evaluated by professionals involved in developing the first survey on the implementation of PBM.

The survey consisted of 10 questions on the following aspects: PBM organization and monitoring, related reports and indicators, patient outcomes and costs. In the last question, participating TSs were asked to specify how the audit was conducted. The requirement for informed consent was waived because the data needed to respond to the survey were all obtained through a retrospective review of data within the TS. Survey responses were provided by the directors of the participating TSs.

TSs were selected among those that had reported high levels of PBM implementation in the previous survey (i.e., those with maximum scores, assigned by awarding one point per affirmative response). All selected institutions were second‐level hospitals [6, 7].

## RESULTS

Participation in the project was 70%, with 14 out of 20 TSs responding. The questions were closed‐ended except for the last one which asked respondents to describe in detail how the audit was conducted and the key points analysed in it.

### Organization and monitoring PBM

**FIGURE 1:**
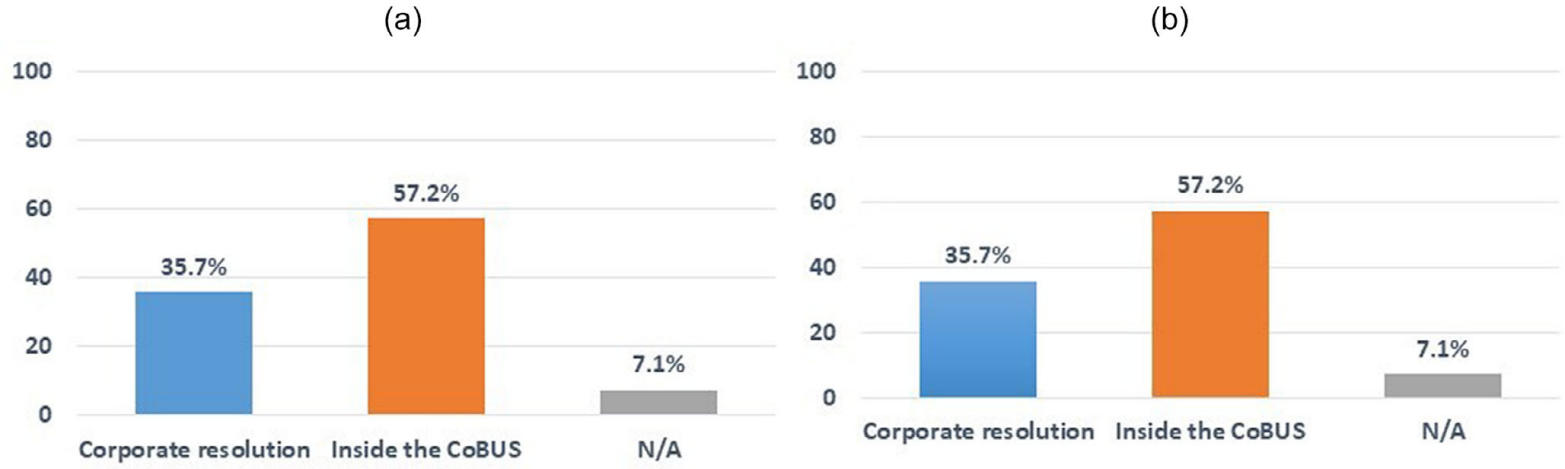
Data on the creation of an internal multidisciplinary working group on patient blood management (PBM) with official formalization of its objectives (a) and the presence of a PBM coordinator with clearly defined responsibilities (b). CoBUS, Committee for the Good Use of Blood; N/A, not applicable (or unresponsive).

As part of organization and monitoring, the creation of an internal multidisciplinary working group on PBM to outline strategies that improve patient safety and optimal clinical outcome, as well as analysing the related results, was formalized within the hospital's Committee for the Good Use of Blood (CoBUS) in 57.2% of responding TSs and through a corporate resolution, for 35.7% (Figure 1a). The same response rates were received for the question on the appointment of a PBM coordinator and a statement on the related responsibilities (Figure 1b).

**FIGURE 2:**
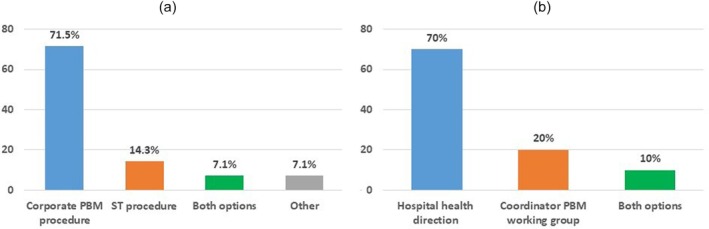
Data on the presence of operational instructions on patient blood management (PBM) within the hospital (a) and on the person responsible for creating such operational instructions (b). TS, transfusion service.

Of the respondents, 71.5% (10 out of 14) reported the presence of a corporate PBM procedure—that is, a set of operational instructions defined in healthcare facility where the transfusion takes place—which describes the development of evidence‐based recommendations for the detection and management of preoperative anaemia and the strategies to manage and preserve the patient's own blood. In 14.3% (2 out of 14), the presence of a PBM procedure was only decided within the TS. Only 7.1% (1 out of 14) reported the presence of both options (a corporate procedure and a TS procedure). One TS indicated the presence of a ‘fast track’ protocol applied only in orthopaedic surgery (Figure 2a).

When an institutional PBM has been developed, responsibility is assigned to the Hospital Health Directorate through the CoBUS in 70% of cases (7 out of 10 TSs). In 20% of cases (2 out of 10), responsibility lies with the coordinator of the internal multidisciplinary PBM working group, while 10% (1 out of 10) report both arrangements (Figure 2b).

### PBM report

**FIGURE 3:**
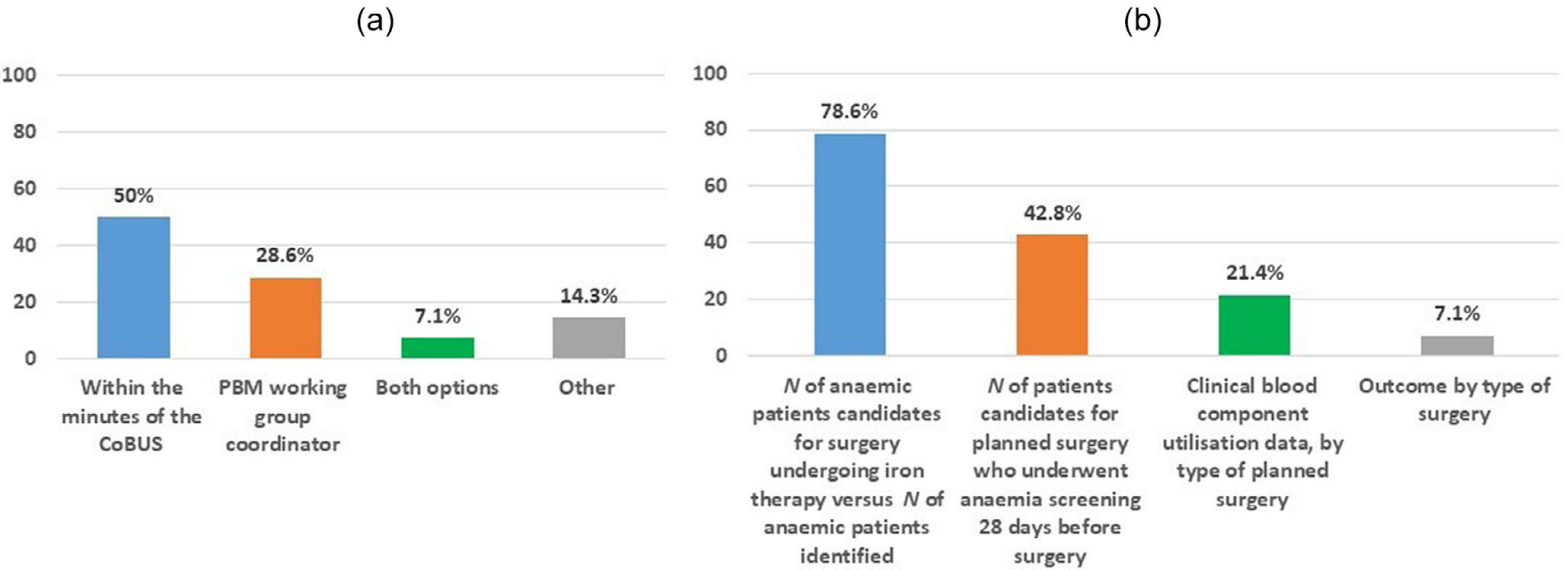
Data on the person responsible for drafting the annual patient blood management (PBM) report (a) and on the indicators contained in the report relating to the number of patients, clinical use of blood components and outcome by type of surgery (b). CoBUS, Committee for the Good Use of Blood; *N*, number.

The PBM report is important for communicating the need for a structured PBM programme to stakeholders. In 50% of cases (7 out of 14), the report is prepared by the CoBUS and included in the official meeting minutes. In 28.6% of cases (4 out of 14), it is prepared by the coordinator of the PBM working group. Both options are reported by 7.1% of respondents (1 out of 14), while 14.3% (2 out of 14) indicate that the anaesthetist and/or orthopaedic specialist is responsible for drafting the report (Figure 3a).

The preparation of an annual PBM report involves several indicators, the most commonly used being the proportion of anaemic surgical candidates who received iron therapy compared to the total number of anaemic patients identified (reported by 78.6% of respondents, 11 out of 14; see Figure 3b).

Other indicators included in the PBM annual report were the following: the number of patients scheduled for elective surgery who underwent anaemia screening at least 28 days prior to the procedure (42.8% of cases, 6 out of 14); data on blood component utilization by type of planned surgery (21.4% of cases, 3 out of 14); and patient outcomes by surgical type (7.1% of cases, 1 out of 14).

### Analysis of the reduction of blood component consumption

**FIGURE 4:**
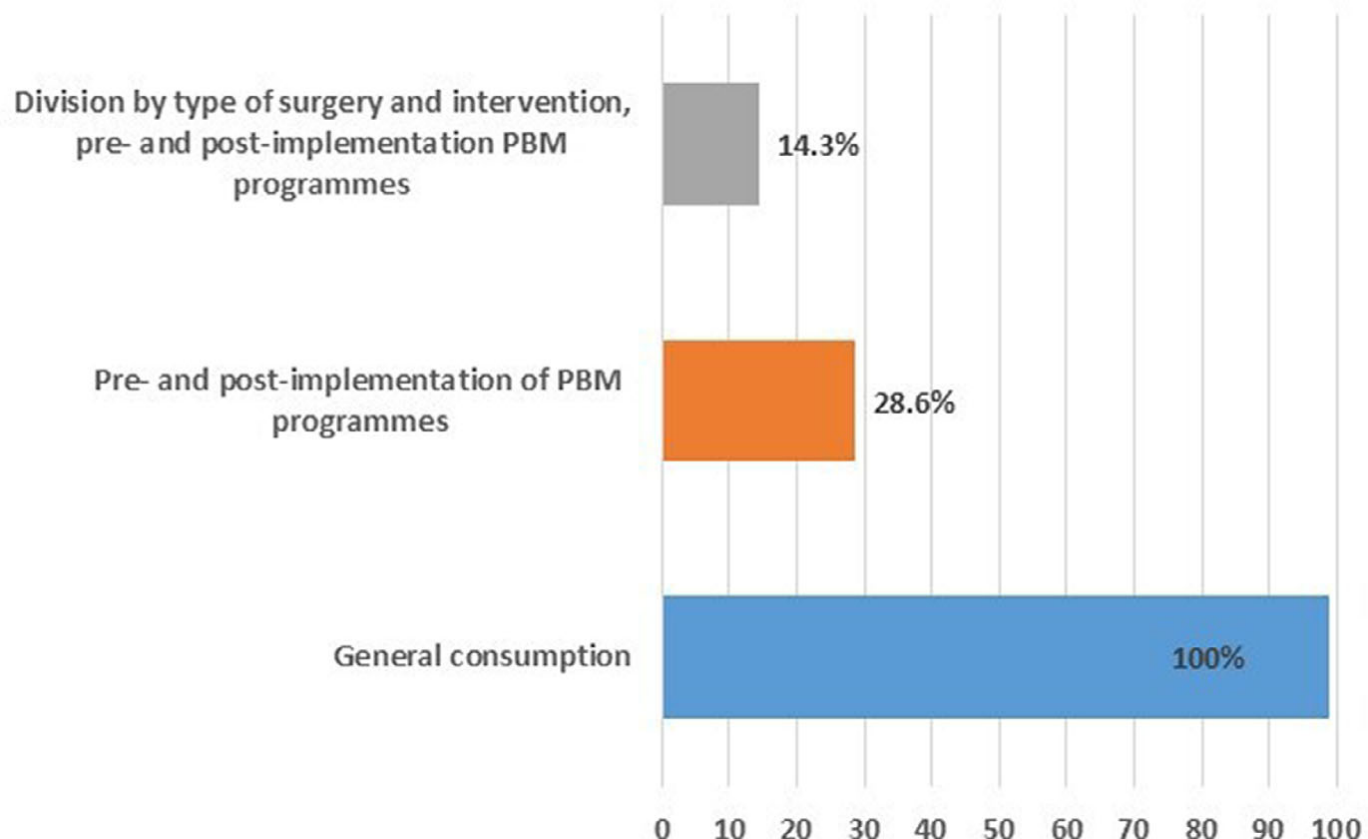
Analysis of the reduction of blood component consumption performed by transfusion services (TSs). General analysis of the blood component consumption for the entire hospital/facility, the reduction of consumption before and after implementation of patient blood management (PBM) programmes and the reduction of consumption by type of surgery and intervention, before and after implementation of PBM programmes.

Regarding the analysis of the reduction of blood component consumption, which covers all blood components, all respondents (100%, 14 out of 14) stated that they only carried out a general analysis of blood component consumption for the entire hospital/ facility (Figure 4).

A comparison of blood component consumption before and after the implementation of PBM programmes was conducted by 28.6% of respondents (4 out of 14). Only 14.3% of respondents (2 out of 14) assessed changes in consumption by type of surgery and intervention during the same period.

### Patient outcomes

Analysis of the reports revealed the difficulty of collecting data on mortality and morbidity outcomes mainly due to the lack of an interface between the existing corporate informatics technologies infrastructures.

**FIGURE 5:**
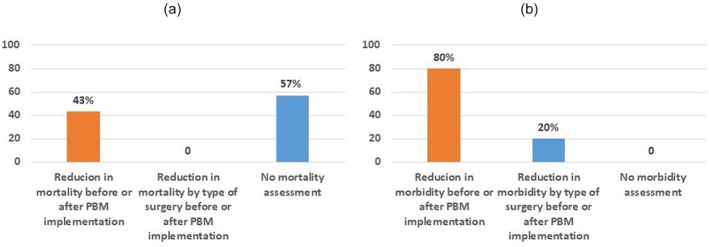
Analysis of the outcome mortality (a) and morbidity (b). PBM, patient blood management.

Figure 5 shows the mortality (Figure 5a) and morbidity (Figure 5b) outcomes before and after PBM implementation by the type of surgery. Within the morbidity outcome, the proportion of patients with prolonged length of stay (LOS), infection rate, adverse transfusion reactions and the rate of hospital readmission were assessed.

The reduction in mortality before and after PBM implementation was made by 6 out of 14 TSs (43%), but none of them reported this value by type of surgery; furthermore, 8 out of 14 (57%) did not analyse the reduction in mortality at all.

On the other hand, the reduction in morbidity before and after PBM implementation was made by 11 out of 14 TSs (80%), and 3 out of 14 (20%) stated that they reported this value by type of intervention. No one said they had not analysed the outcome of morbidity.

### Analysis of related PBM costs

Regarding the analysis of PBM‐related costs, the items evaluated were laboratory tests, iron therapy, haemostatic drugs and viscoelastic monitoring tools.

**FIGURE 6:**
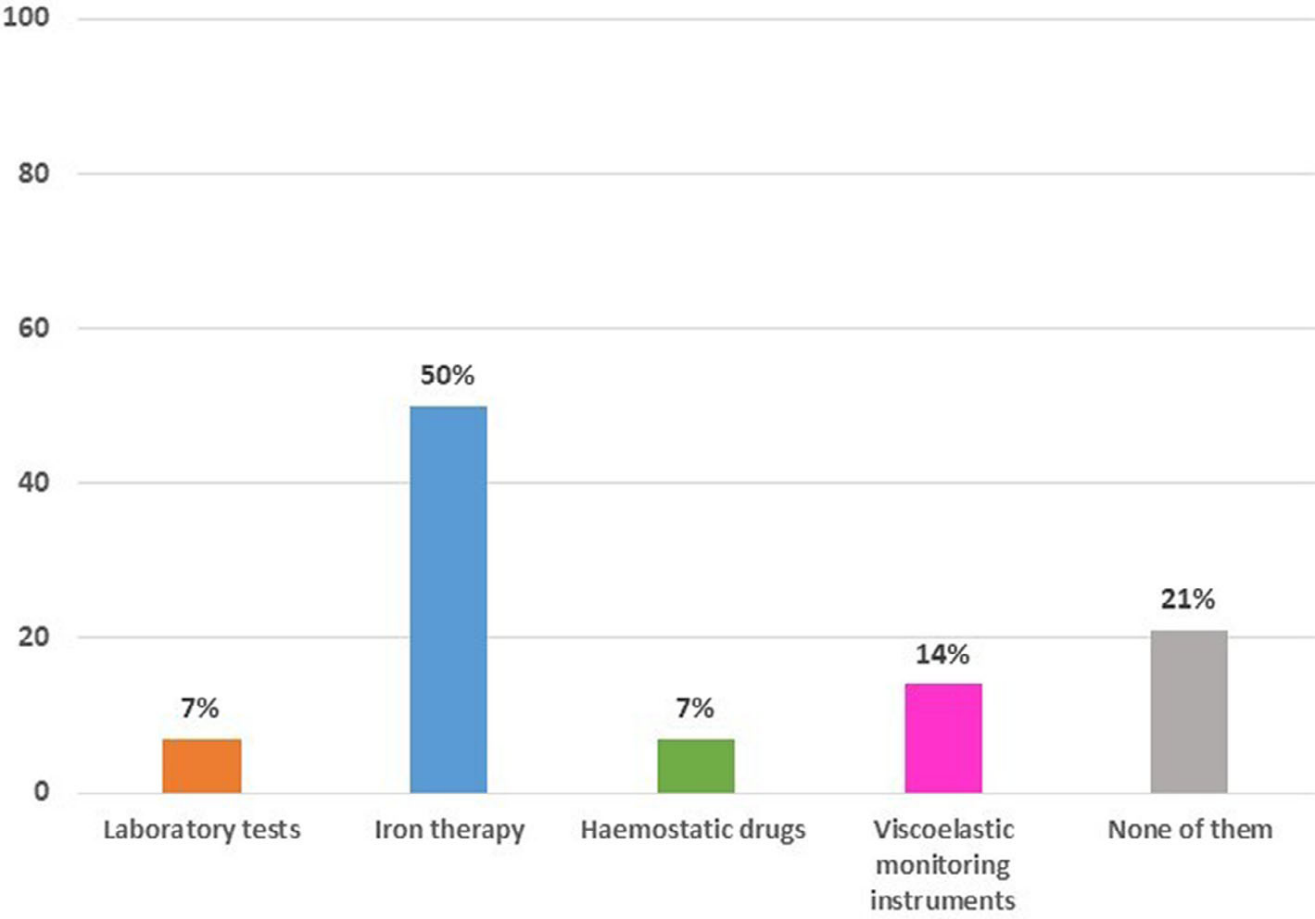
Elements considered for patient blood management (PBM) related costs (percentage value): Laboratory tests, iron therapy, haemostatic drugs and viscoelastic monitoring instruments.

Figure 6 shows the rates of the analysis of PBM‐related costs by respondents to the survey.

Only 1 out of 14 (7%) said they considered the costs of laboratory tests; the same applies to haemostatic drugs. Only 2 out of 14 (14%) stated that they considered the costs of viscoelastic monitoring tools; 7 out of 14 (50%) considered the costs of iron therapy, and 3 out of 14 TSs (21%) did not carry out any kind of economic evaluation.

### Planning annual PBM audit

Regarding the request to clarify how the annual audit on PBMs is carried out, no TS provided an answer; therefore, it was not possible to analyse this aspect. Clinical auditing of PBM is probably not standardized with fixed appointments, and there is no real analysis of risks and possible corrective or improvement actions.

### Summary table of results

A summary table of results is presented to facilitate reading of the figures (Table 1).

**TABLE 1:** Summary table of results.

	Corporate resolution	Inside the CoBUS	N/A
PBM working group creation	35.7%	57.2%	7.1%
PBM working group coordinator	35.7%	57.2%	7.1%

	Corporate resolution	Inside the TS	Both	Other
PBM procedure	71.5%	14.3%	7.1%	7.1%

	Hospital health direction	Coordinator PBM working group	Both options
PBM procedure responsibility	70%	20%	10%

	Within the CoBUS	PBM working group coordinator	Both options	Other
PBM annual report realization	70%	20%	10%	4.3%

	Within the CoBUS	PBM working group coordinator	Both options	Other
PBM annual report realization	70%	20%	10%	14.3%

	No. of anaemic patients' candidates for surgery undergoing iron therapy versus no. of anaemic patients identified	No. of patient candidates for planned surgery who underwent anaemia screening 28 days before surgery	Clinical blood component utilization data, by type of planned surgery	Outcome by type of surgery
Indicators in PBM annual report	78.6%	42.8%	21.4%	7.1%

	General consumption	Pre‐ and post‐implementation of PBM programmes	Division by type of surgery and intervention, pre‐ and post‐implementation PBM programmes
Reduction of blood component consumption	100%	28.6%	14.3%

	Reduction in mortality before or after PBM implementation	Reduction in mortality by type of surgery before or after PBM implementation	No mortality assessment
Outcome mortality	43%	–	57%

	Reduction in morbidity before or after PBM implementation	Reduction in morbidity by type of surgery before or after PBM implementation	No morbidity assessment
Outcome morbidity	80%	20%	–

	Laboratory tests	Iron therapy	Haemostatic drugs	Viscoelastic monitoring instruments
Analysis of PBM‐related cost	7%	50%	7%	14%

Abbreviations: CoBUS, Committee for the Good Use of Blood; N/A, not applicable; PBM, patient blood management; TS, transfusion service.

## DISCUSSION

The overarching goal of the PBM strategy is to improve global blood health by optimizing the management of patients' own blood. In the perioperative setting, this involves addressing the full continuum of care before, during and after surgery through appropriate treatment of anaemia, identification of bleeding risk, prevention and treatment of bleeding and improved storage and conservation strategies, resulting in a comprehensive evaluation of each case and all possible treatment alternative [9]. This patient‐centred approach improves patient outcomes and safety, reduces transfusion‐related complications and, as a secondary benefit, contributes to blood supply sustainability [10]. For this reason, all healthcare providers should aim for continuous implementation of PBM programmes.

This is the second survey on PBM implementation in Italy. The results show that TSs are attempting to implement PBM measures, but this implementation is not uniform or widespread.

At the organizational level, there is a need for a regional resolution and/or regulatory action to create a preoperative checklist to identify preoperative anaemia and bleeding risk, and to establish a dedicated clinic staffed by a transfusion medicine manager and an expert in haemostasis and thrombosis [11]. The drafting of an integrated multidisciplinary protocol, shared among the different specialities involved (general and specialty surgery, orthopaedics and traumatology, gynaecology and obstetrics, anaesthesia and resuscitation, transfusion medicine, clinical risk, haemostasis and thrombosis, clinical pathology laboratory and haematology) can be useful for the development of specific process and outcome indicators involving individual surgical units.

Figure 1 shows that the creation of an internal multidisciplinary working group for the implementation of specific PBM programmes in accordance with the Decree of the Ministry of Health (2 November 2015), the definition of its objectives and the formalization of the appointment of a PBM coordinator are activities mostly carried out within the CoBUS.

Although diagnostic‐therapeutic pathways are generally recognized, their implementation is inconsistent across hospitals (Figure 2).

A shared multidisciplinary operational guideline across all medical specialties supports the stratification of surgical procedures by bleeding risk, enabling the early identification of patients who require preoperative optimization. At the same time, it ensures that all surgical patients can benefit from the principles of PBM, regardless of their individual risk profile. Enrolled patients should be evaluated for any comorbidities, bleeding risk related to the type of surgery and patient‐dependent bleeding risk, at least 28–30 days prior to the scheduled surgery. Any case of anaemia identified in the preoperative phase must be appropriately diagnosed and treated, as recommended by the CNS in the anaemia clinic, preferably in charge of transfusion medicine, unless otherwise organized by the hospital. Postoperative assessment should also preferably be entrusted to the transfusion medicine department, depending on the patient's clinical condition [12].

The development of a business plan must consider not only the available guidelines and scientific evidence on the subject but also the evaluation of retrospective data collected through preliminary audits, an adequate analysis of the skills of available human resources and the equipment available at the local level. To this end, it is essential to identify the leadership of the multidisciplinary and multiprofessional team and map the main stakeholders, as well as make a rational choice of the effectiveness and process indicators [13].

While the annual PBM report is an essential tool, it requires considerable data coordination, stakeholder input and institutional support. The annual report also provides useful data for evaluating the implemented strategies and initiatives, facilitating the monitoring of activities and contributing to more efficient and continuously improving PBM management. The preparation of an annual PBM report involves several indicators, the most important of which is the number of anaemic patients who are candidates for surgery and undergo iron therapy compared to the number of anaemic patients identified (Figure 3).

The correct implementation of a PBM programme should be assessed for individual surgical and/or medical specialties. Monitoring through KPIs is essential for evaluating the correct implementation of PBM while also capturing the clinical use of blood products by individual hospital units. These should be chosen to represent all stages of PBM application, from pre‐admission to patient discharge, while assessing the clinical use of blood products by the individual operating units involved [14].

About the analysis of the reduction in blood component consumption, the TSs only carried out a general analysis of blood component consumption for the entire hospital/ facility (Figure 4). However, PBM programmes offer several strategies to improve haematopoiesis and prevent unnecessary blood loss, particularly in case of major surgery (which is commonly associated with critical blood loss) [15]. PBM could certainly be more effective in patients undergoing major surgery, and it would be interesting to evaluate its effectiveness among different healthcare professionals.

Analysis of the reports received from the second survey revealed the difficulty in collecting data on mortality and morbidity due to communication gaps between corporate information technology infrastructures: the reduction in morbidity before and after implementation appears to be more significant than mortality (Figure 5). The most relevant clinical outcomes include mortality and serious morbidity events, but of course, the analysis of outcomes is more complex than simply analysing haemoglobin values or infusion rates [16].

Finally, the analysis of PBM‐related costs (Figure 6) focused primarily on iron therapy, but a more comprehensive evaluation should include comparisons with the pre‐implementation period particularly regarding costs related to anaesthesiology assessments, preoperative testing, medication administration, LOS in the hospital and follow‐up visits [17]. Reducing the cost of managing patients who are candidates for scheduled surgery represents the main expectation for healthcare management's involvement in a standardized PBM programme.

A nationwide study conducted in the United Kingdom by Klein et al. in 2016 [18] showed that anaemia is common prior to cardiac surgery and that patients with anaemia have a significantly higher risk of death after such surgery. Another single‐centre study conducted in Italy also showed a strong association between preoperative anaemia and increased mortality, stroke and other serious morbidities [19].

Other obstacles to the implementation of PBM were examined by Filipescu et al. in 2025, who, in a survey conducted among anaesthetists, highlighted the low frequency of institutional standard operating procedures for this purpose [20].

Finally, the latest WHO guidelines on the implementation of PBM [21] report the result of a collaboration among multiprofessional and multidisciplinary international experts dedicated to improving patient outcomes, safety and quality of care. The document identifies elements for national/jurisdictional implementation of PBM involving the most relevant stakeholders and examples of PBM implementation for specific patient populations.

In conclusion, despite the trend towards high‐level practices, and despite the WHO publishing a new document in 2021 emphasizing the urgent need to bridge the gap in awareness and implementation of PBM by announcing an upcoming initiative to develop specific guidelines, few European countries currently have national and standardized guidelines of PBM.

In this work, having analysed mainly the organizational elements of the PBM and identified the main critical issues, we can recommend greater awareness among health management and hospital management with a view to centralizing pre‐admissions, involving regional clinical risk representatives, and suggesting continuous interaction between clinicians, also with a view to obtaining consistent assessments of results.

The two main limitations of this study are that it only considered certain aspects of PBM and only involved some Italian TSs, namely those who had previously stated that they considered PBM to be an approach worth following. This introduces a potential selection bias, as these centres may already have more advanced implementation practices, limiting the generalizability of the results to all Italian TSs.

However, this second survey allowed a comparison between the main critical issues and the advantages that emerged, which led to the creation of a policy document that, reflecting the realities on the ground, sets out some important recommendations for the implementation of an advanced PBM system to be applied throughout the country, such as the identification of an operational protocol for the correct management of patients according to a PBM programme, an example of a PBM working group, monitoring of indicators, and the need for continuous education of healthcare professionals.

*Data processing*: The authors confirm that no identifiable information has been disclosed and that the data processing has been carried out in accordance with the relevant data protection regulations. This work represents an innovative analysis.

## CONFLICT OF INTEREST STATEMENT

The authors declare no conflicts of interest.

## Supporting information


**Supporting Information S1.** Survey on patient blood management's organizational aspects.

## Data Availability

Data sharing not applicable to this article as no datasets were generated or analysed during the current study.
