# Supplementary material for: Organizational monitoring of patient blood management implementation: Results of an Italian national survey
Source: Vox Sang. 2026 May 15;121(7):1041–9. doi: 10.1111/vox.70267 (PMC13356996; doi:10.1111/vox.70267)
Supplement: Supplementary file 1 — Supporting Information S1. Survey on patient blood management's organizational aspects. [file VOX-121-1041-s001.docx]

**Supplementary Content S1.** Survey on Patient Blood Management’s organisational aspects

| 1. In the organizational model you have adopted, the creation of an internal multidisciplinary working group on PBM and the declination of its objectives has been formalized: | - through corporate resolution; - within the CoBUS. |
| --- | --- |
| 2. The appointment of a PBM coordinator and the definition of related responsibilities have been formalized: | - through corporate resolution; - within the CoBUS. |
| 3. In the organizational model you have adopted, provision has been made for the following: | - a corporate PBM procedure within their structure; - a PBM procedure within the ST; - both options; - other. |
| 4. Where a company procedure on PBM has been developed in the organizational model, responsibility has been placed on: | - Hospital Health Direction through the CoBUS; - coordinator of the internal multidisciplinary working group on PBM; - both options. |
| 5. An annual PBM Report is prepared: | - within the minutes of the CoBUS; - PBM working group coordinator, - both options, - other. |
| 6. The preparation of an annual PBM Report includes: | - clinical blood component utilization data, by type of planned surgery; - outcome by type of surgery; - number of patients who were candidates for planned surgery and who underwent anaemia screening 28 days before surgery; - number of anaemic patients who are candidates for surgery undergoing iron therapy versus the number of anaemic patients identified. |
| 7. For the analysis of the reduction in blood component consumption, the following is calculated: | - general analysis of the blood component consumption for the entire hospital/ facility; - reduction of consumption pre- and post-implementation of PBM programs; - reduction of consumption by type of surgery and intervention, pre- and post-implementation PBM programs. |
| 8. Related to the following patient outcome “reduction in hospital mortality”: | - reduction in mortality before and after PBM implementation; - reduction in mortality before and after PBM implementation by type of surgery; - no mortality assessment. |
| 9. Related to the following patient outcome “morbidity”: | - reduction in morbidity before and after PBM implementation; - reduction in morbidity before and after PBM implementation by type of surgery; - no morbidity assessment |
| 10. For the analysis of related PBM costs, an assessment was made on: | - laboratory tests; - iron therapy; - haemostatic drugs; - viscoelastic monitoring instruments; - none of them. |
| 11. Regarding the scheduling of the annual PBM Audit, please make explicit how the audit will be conducted and the items examined. | |

CoBUS: Committee for the Good Use of Blood; PBM: patient blood management; ST: Transfusion Service.
